# Supplementary material for: Scutellaria petiolata Hemsl. ex Lace & Prain (Lamiaceae).: A New Insight in Biomedical Therapies
Source: Antioxidants (Basel). 2022 Jul 26;11(8):1446. doi: 10.3390/antiox11081446 (PMC9331036; doi:10.3390/antiox11081446)
Supplement: Supplementary file 1 [file antioxidants-11-01446-s001.zip › antioxidants-1779807-supplementary.pdf]

Supporting Information

Scutellaria petiolata Hemsl. ex Lace & Prain (Lamiaceae).: A New Insight in Biomedical Therapies

Sidra Mubin[1], Najeeb Ur Rehman[2], Waheed Murad[3], Muddaser Shah[2,3*], Ahmed Al-Harrasi[2*] and Rabia Afza[1*]

[1]Department of Botany, Hazara University Mansehra, Mansehra 21310, Pakistan; shahhu123@gmail.com (SM); ethnopk@gmail.com(RA).

[2]Natural and Medical Sciences Research Center, University of Nizwa, P.O. Box 33, Birkat Al Mauz, Nizwa 616, Oman; najeeb@unizwa.edu.om (N.U.R)

[3]Department of Botany, Abdul Wali Khan University Mardan, Mardan 23200, Pakistan; waheedmurad@awkum.edu.pk (W.M)

*Correspondance: muddasershah@awkum.edu.pk (MS) aharrasi@unizwa.edu.om(A.A.H), rabiaafza@hu.edu.pk(RA),

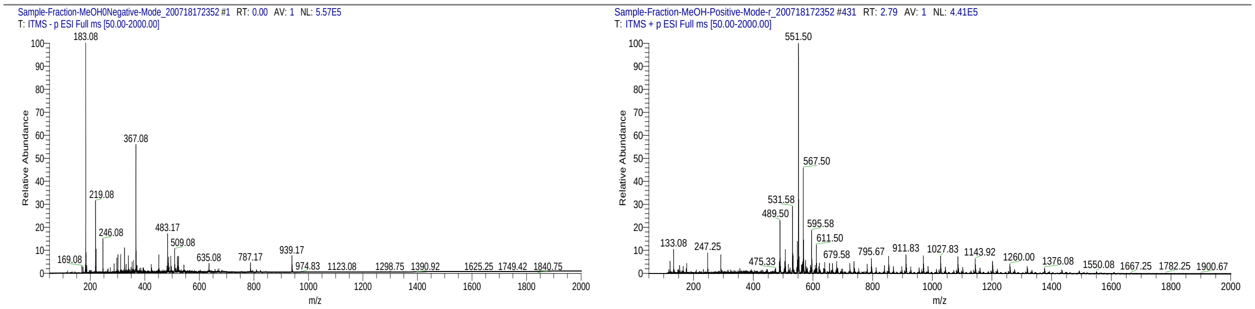

A) **B)**

Figure S1. Full-scan ESI-LC-MS mass spectrum of the SPM extract of *S. petiolata*. (A) negative ionization mode and (B) positive ionization mode.

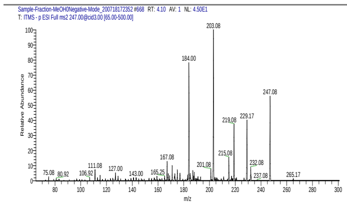

1

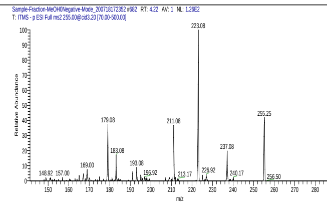

2

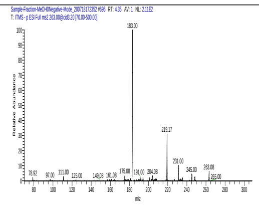

3

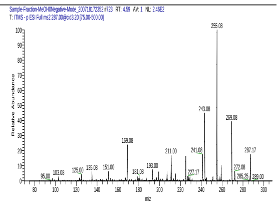

4

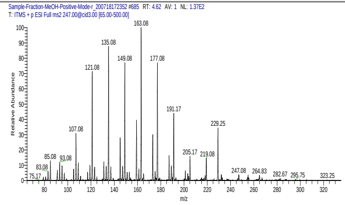

5

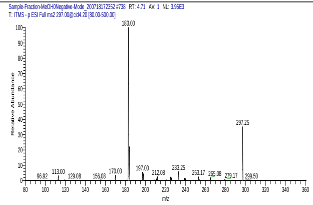

6

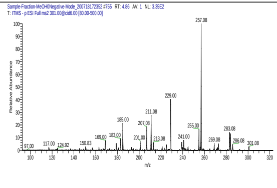

7

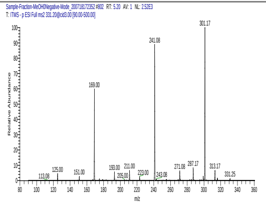

8

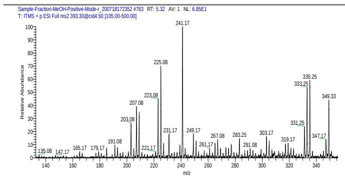

9

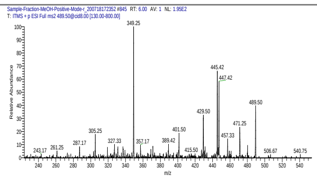

10

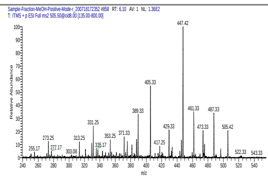

11

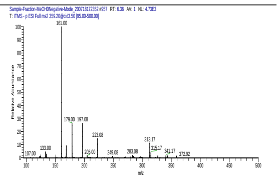

12

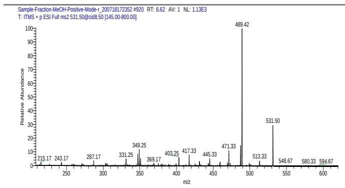

13

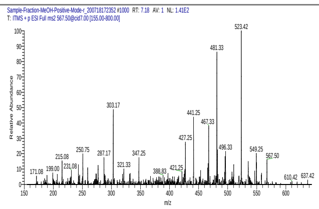

14

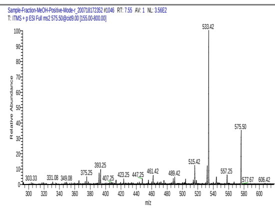

15

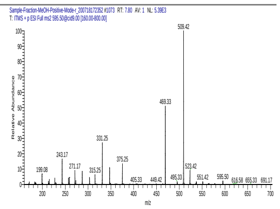

16

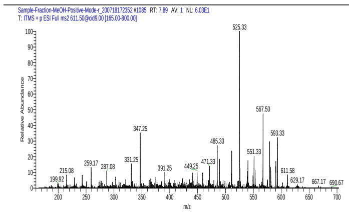

17

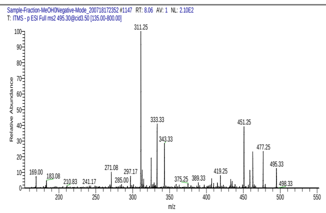

18

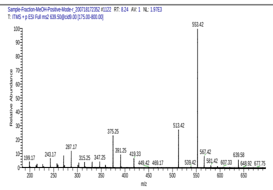

19

Figure S2: Mass spectrum of the respective tentatively identified chemical ingredients via ESI-LC-MS analysis in the active MeOH extract of *S. petiolata*.
